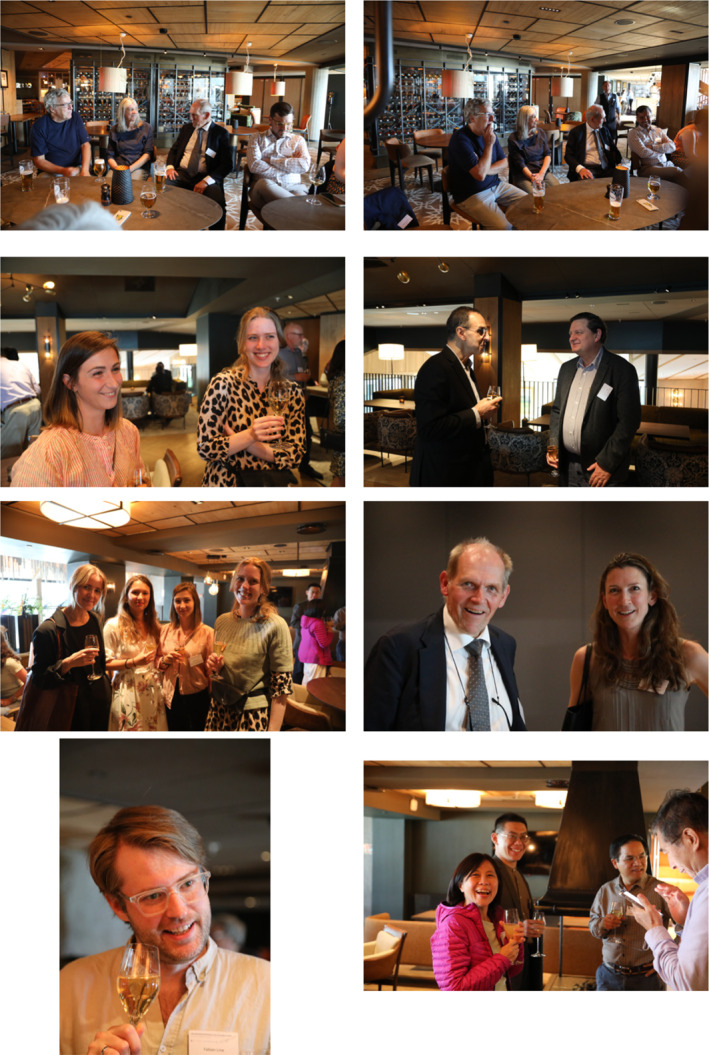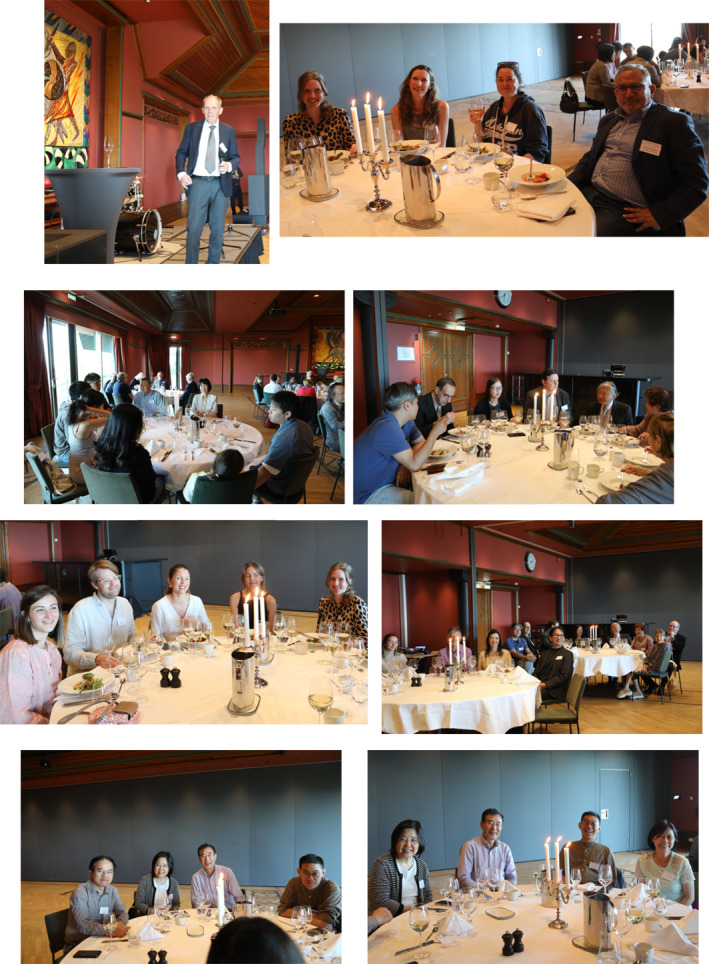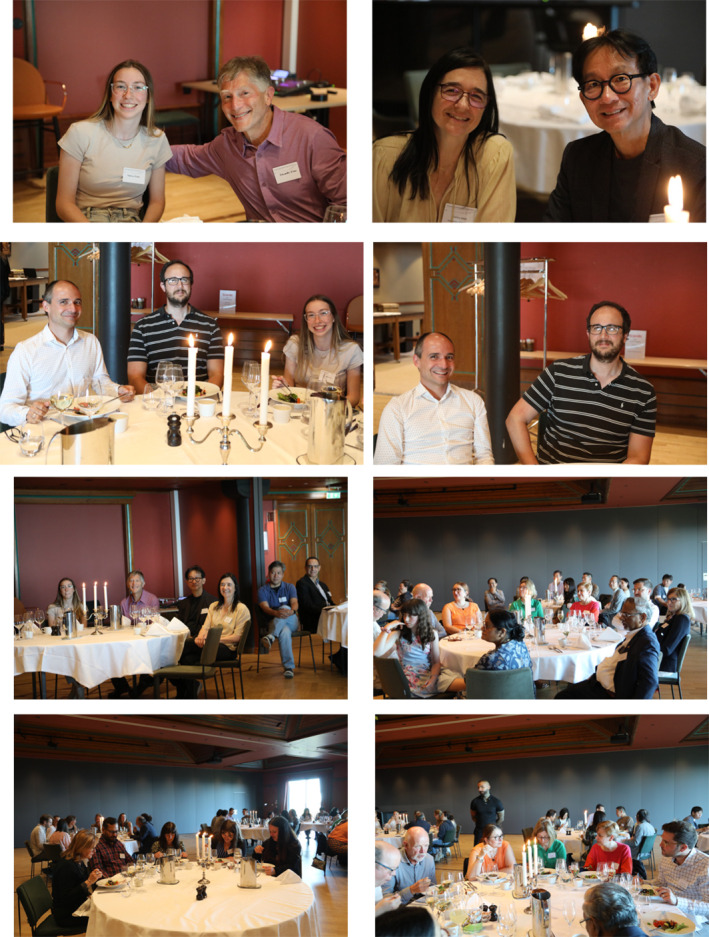# The 12th international workshop on the CCN family of genes in pictures

**DOI:** 10.1002/ccs3.12051

**Published:** 2024-11-13

**Authors:** Annick Perbal

**Affiliations:** ^1^ International CCN Society Nice France

From June 20th to June 23rd, the 12th International workshop on the CCN Family of Genes has been held at the **Scandic Holmenkollen Park Hotel, OSLO–Norway**


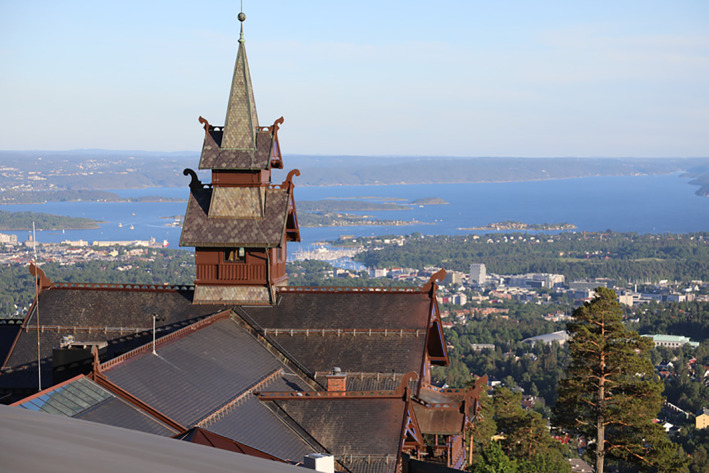


**Organizers**

Professor Håvard Attramadal and Dr. Vivi T. Monsen, Oslo University Hospital

**Co‐organizers**

Professor Bernard Perbal and Annick Perbal, International CCN Society, Nice France

The workshop has been scientifically and socially very successful.

Since the previous meeting held in Nice in 2022, it has been opened to different fields.

This year, Dr. Katia Scotlandi from Bologna, Italy, has been selected to be the 9^th^ ICCNS Awardee.


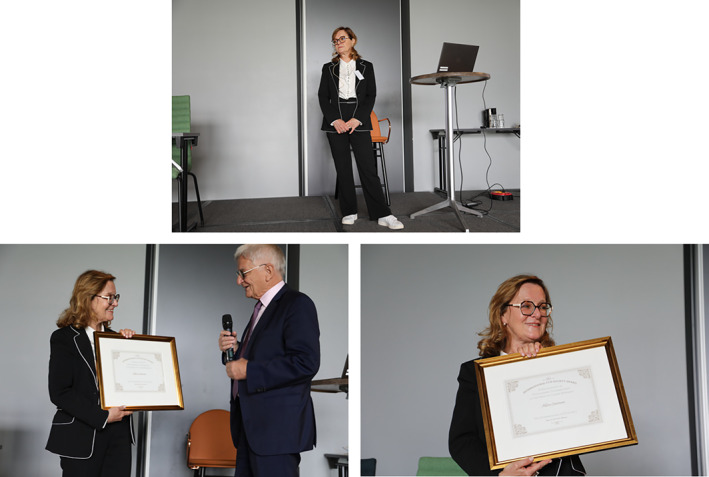



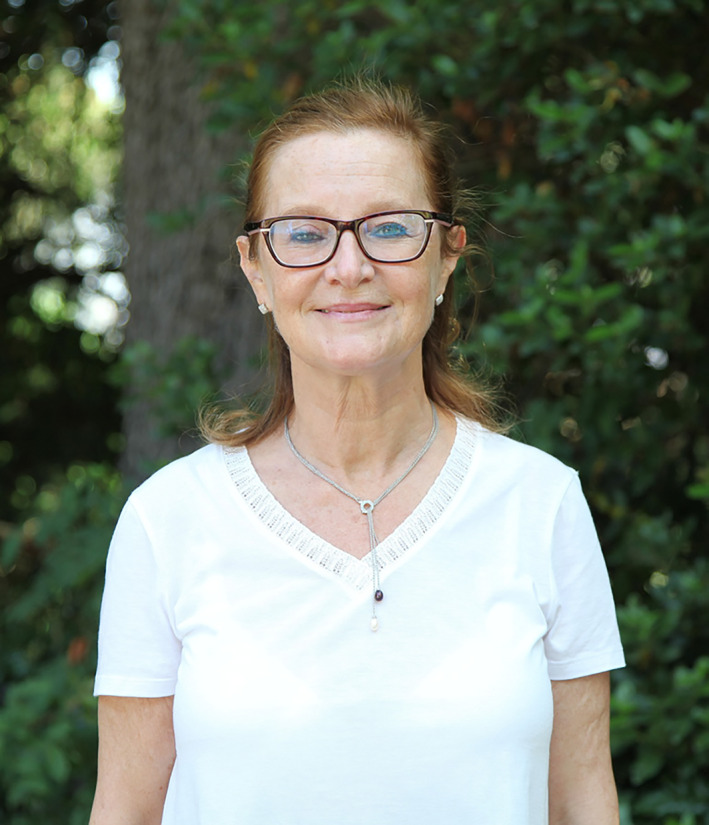


Dr. Katia Scotlandi has long been committed to advancing research and scientific interest in the field of IGF and insulin system. Her scientific group has demonstrated the importance of the related signaling pathway in sarcomas, particularly in the Ewing sarcoma and participated in the development of rationale strategies to inhibit IGF1R‐mediated signaling at preclinical level. She has also highlighted the role of the insulin receptor in the rapid development of resistance to antibodies targeting IGF1R. More recently, she has introduced the concept that the RNA‐binding protein IGF2BP3 may regulate the cell sensitivity to anti‐IGF1R agents. In addition she has significantly contributed hard to the identification of novel biomarkers of risk and prognosis, including CCN3, as well as of new therapeutic targets for these tumors. More recently, she has developed a platform for sequencing and establishment of complex preclinical models to accelerate our understanding of bone sarcomas.


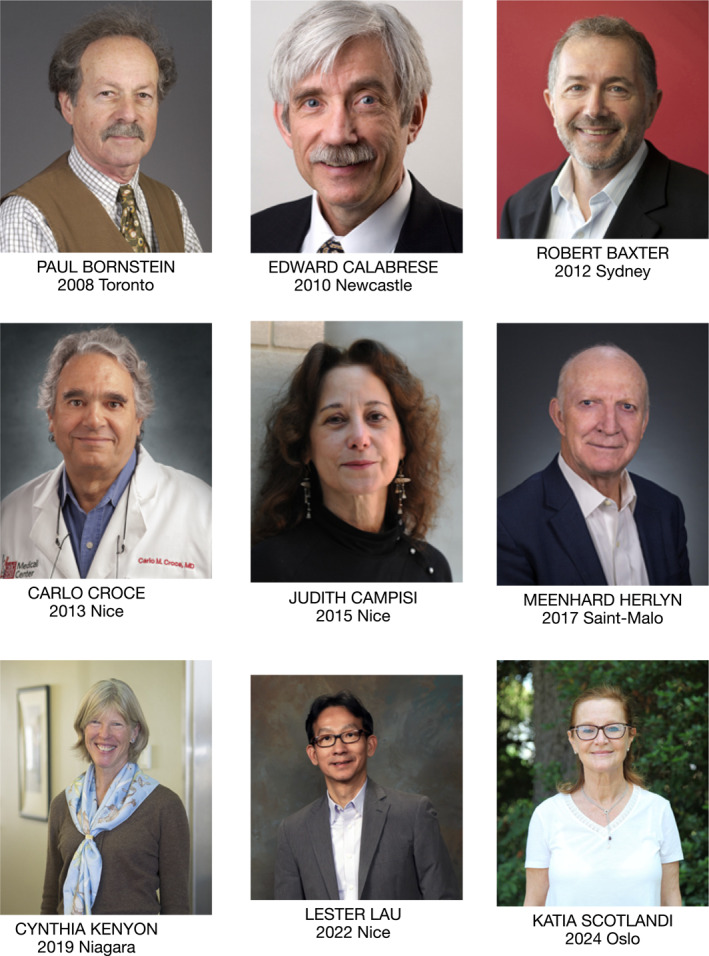


**Listing of the ICCNS awardees**

**Program**

**T** **hursday, June 20**

**9:00 Registration begins**

**10:30–11:30 Business meeting for the JCCS Editorial Board.**

**11:45–13:00 Lunch**

**Workshop Opening**


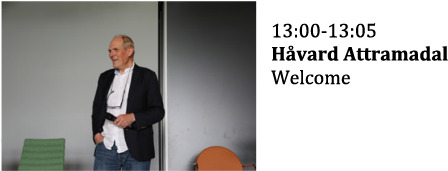



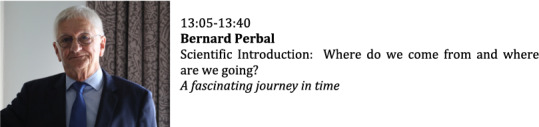


**Session I**

**ECM Proteins in Cell Communication and Signaling**

**Chairs: Brahim Chaqour and Vivi T. Monsen**


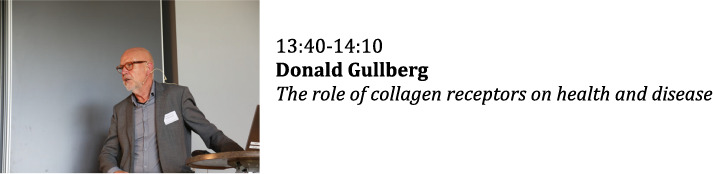



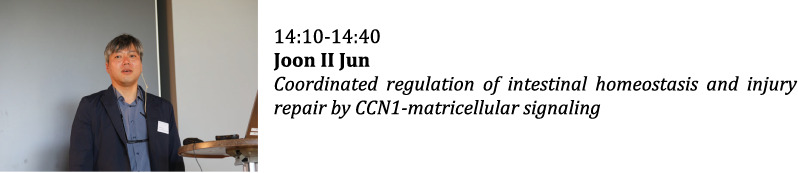


**14:40–15:10 Coffee Break**


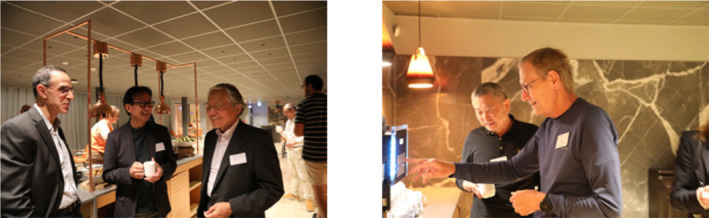



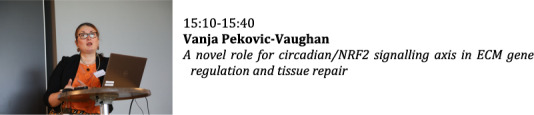



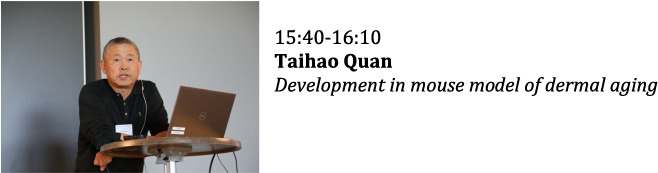


**18:30 Welcome reception and Dinner with Live Music at Scandic Holmenkollen Park Hotel**

**Session II**

**Vascular Development and Pathophysiology**

**Chairs: Lester Lau and Håvard Attramadal**


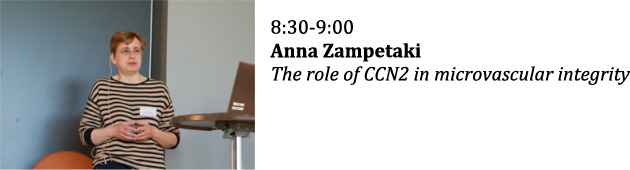



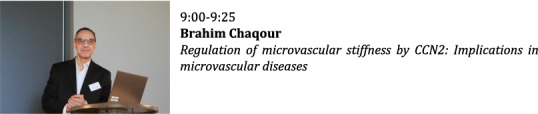



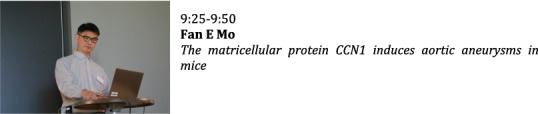


**9:50–10:20 Coffee Break**


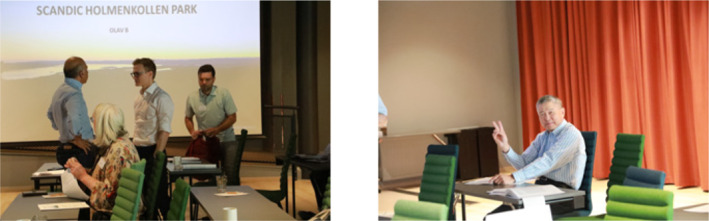



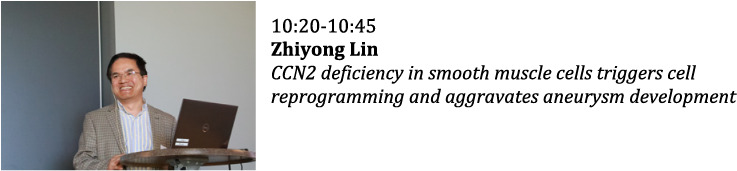



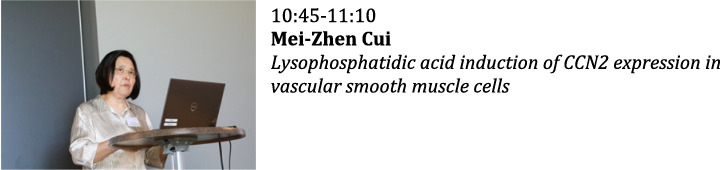


**Session III**

**Mechanisms of Diseases: Fibrosis and The Matrix**

**Chairs: George Bou‐Gharios and Satoshi Kubota**


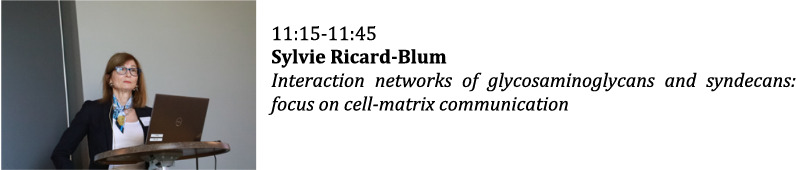



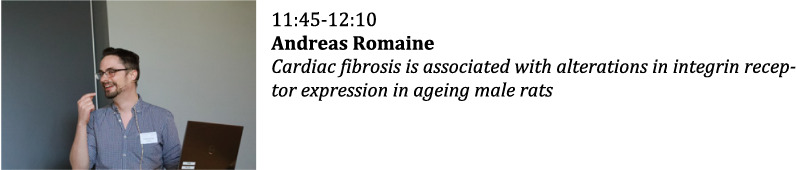


**Lunch 12:10–13:30**


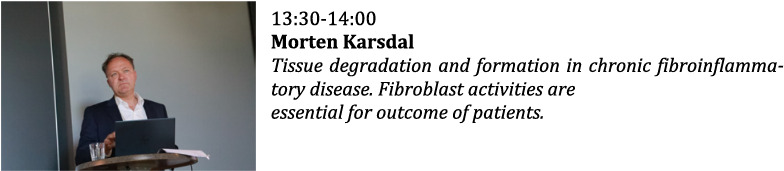



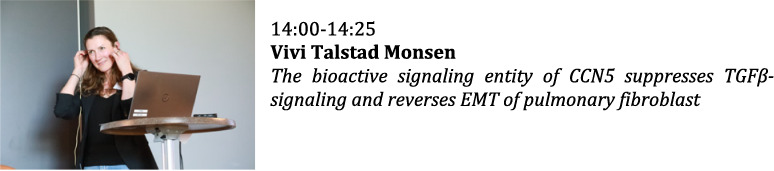



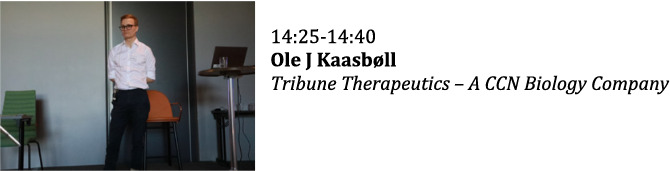



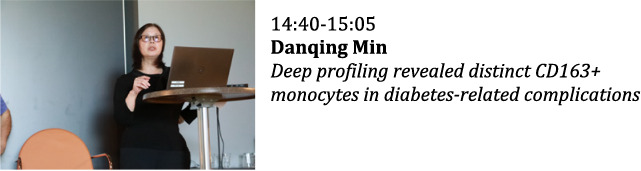



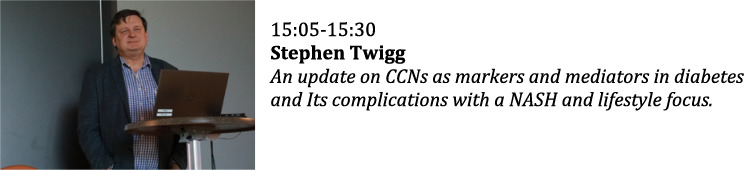


**Boat Trip on the Oslo Fjord with dinner**


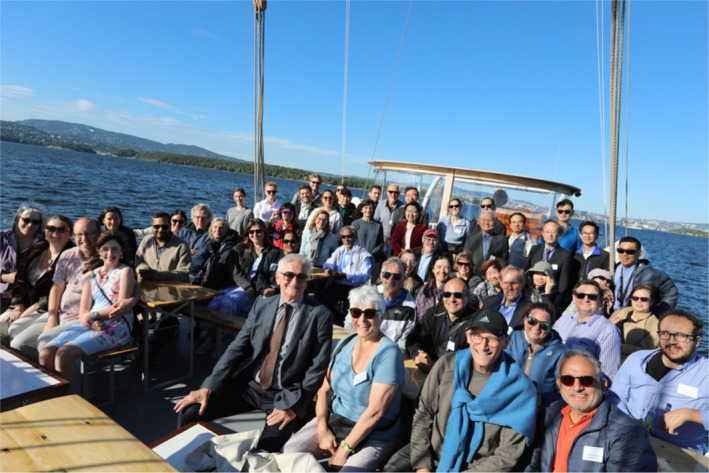


**Saturday, June 22**

**Session IV**

**Tissue Development and Homeostasis**

**Chairs: Blandine Poulet and Bernard Perbal**


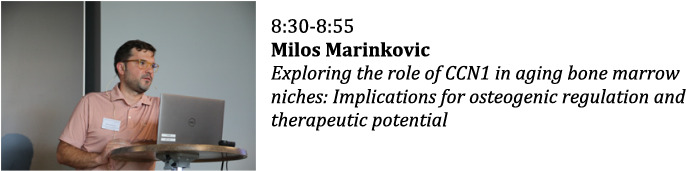



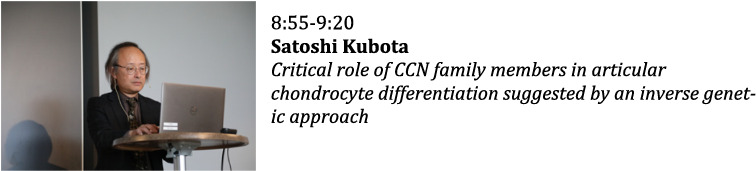



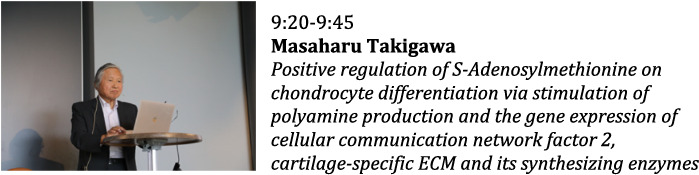


**9:45–10:15 Coffee Break**


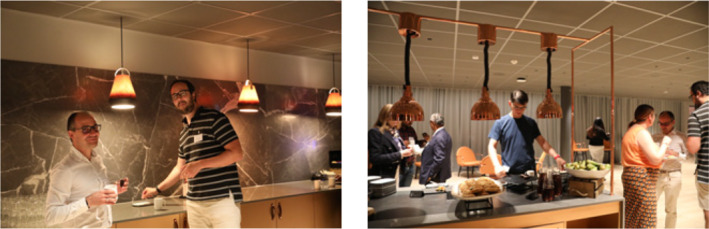



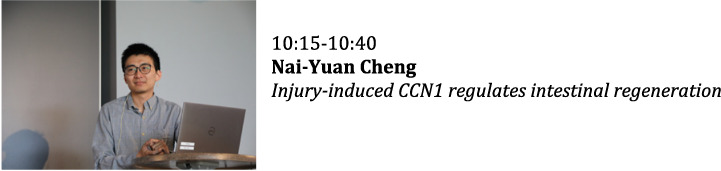



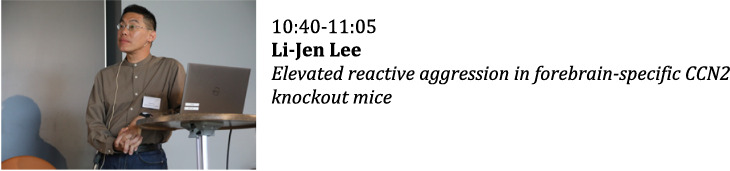



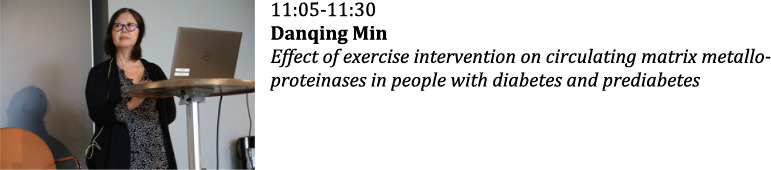



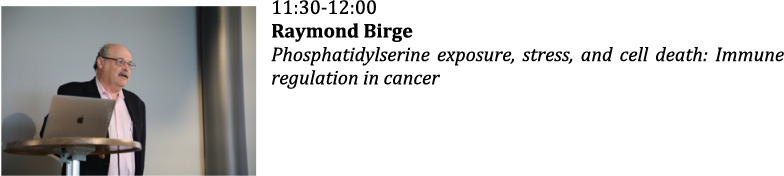


**Lunch 12:00‐13:00**

**Session V**

**Mechanisms of Disease: Cancer and the Matrix**

**Chairs: Stephen Twigg and Ray Birge**


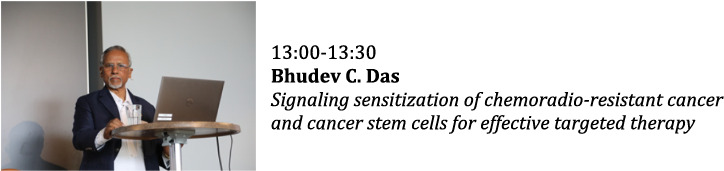



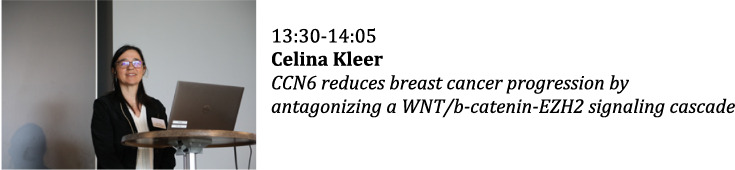


**14:05–14:30 Coffee Break**


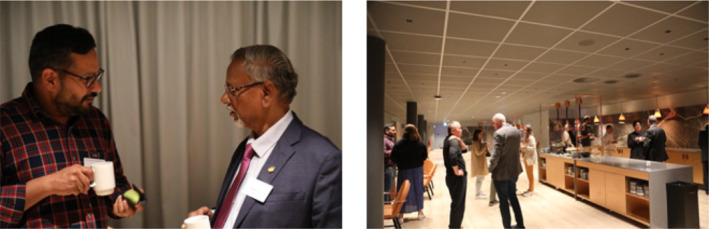



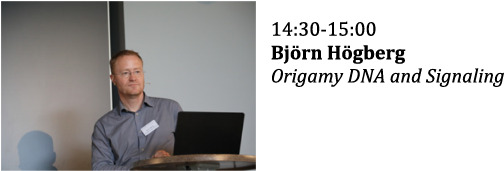



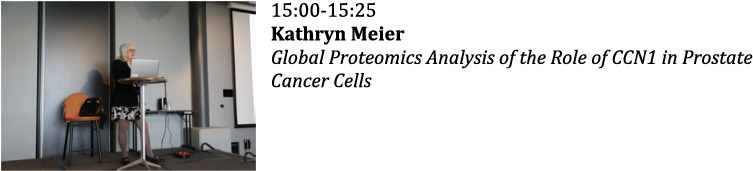



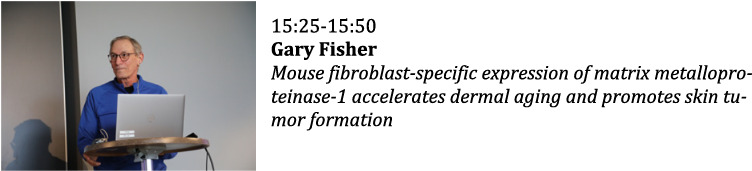


**Free evening**

**Special JCCS Executive Board Meeting**

**Sunday, June 23**

**Session VI**

**Chair: Bernard Perbal**

8:45–9:00

**Bernard Perbal**

ICCNS Award Presentation


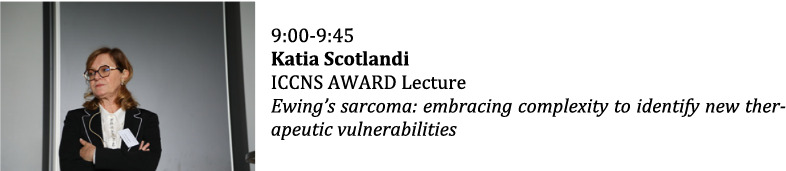


**Educational Lecture**


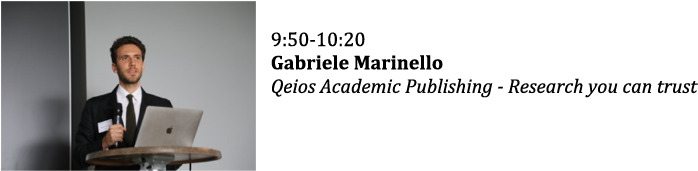


**10:20–10:45 Coffee Break**


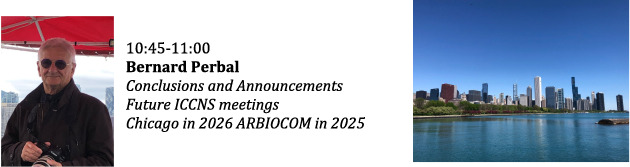



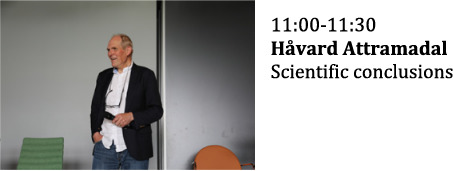


**12:00 Lunch** Departure of the participants